# Impact on Growth and Feed Availability from Including Jack Mackerel (*Trachurus japonicas*) Meal in Rockfish (*Sebastes schlegeli*) Feeds Which Otherwise Replace Fish Meal with Chicken By-Product Meal

**DOI:** 10.3390/ani14081203

**Published:** 2024-04-17

**Authors:** Ran Li, Sung Hwoan Cho

**Affiliations:** 1 Department of Convergence Study on the Ocean Science and Technology, Korea Maritime and Ocean University, Busan 49112, Republic of Korea; ytliran@126.com; 2 Division of Convergence on Marine Science, Korea Maritime and Ocean University, Busan 49112, Republic of Korea

**Keywords:** rockfish (*Sebastes schlegeli*), chicken by-product meal, jack mackerel meal, feed enhancer, economic profit index

## Abstract

**Simple Summary:**

Fish meal is commonly used as the main protein source in fish feeds, but its high price has restricted the use of fish meal. Thus, looking for a substitute with an inexpensive and sustainable source for fish meal in fish feeds is highly needed. Nevertheless, replacement of fish meal with an alternative source in fish feeds commonly reduces the palatability of diets, leads to reduced feed intake, and lowers growth performance. Our previous study reported that up to 20% of fish meal could be substitutable with chicken by-product meal in the feed of rockfish without deteriorating their growth and feed intake. This study evaluates the impact on growth and feed availability from including jack mackerel meal in rockfish diets which otherwise replace 20% fish meal with chicken by-product meal. With fish fed a 55% fish-meal-based diet, the inclusion of 60 and 80% jack mackerel meal in low-fish-meal diets achieved superior weight gain, specific growth rate, and feed consumption. Furthermore, compared to an 80% inclusion level, the slightly but not significantly higher economic profit index was obtained in rockfish fed the low-fish-meal diet with 60% jack mackerel meal in diets. Therefore, the manipulation of jack mackerel meal as feed enhancer in the low-fish-meal diet, which otherwise replaces 20% fish meal with chicken by-product meal, could improve the growth performance and feed consumption of rockfish.

**Abstract:**

This study was conducted to elucidate the impact on the growth and feed availability of rockfish (*Sebastes schlegeli*) from including jack mackerel meal (JMM) in feeds which otherwise substitute 20% fish meal (FM) with chicken by-product meal (CBM). Six formulated feeds were designed to be isonitrogenous and isolipidic. Specifically, 55% FM was included in the control (Con) diet. In the Con diet, 20% FM was substituted by CBM, and then the graded levels (0, 20, 40, 60, and 80%) of JMM were included instead of FM, named as the C20J0, C20J20, C20J40, C20J60, and C20J80 diets, respectively. Five hundred and forty juvenile rockfish (initial weight of 11.2 g) were assigned to 18 tanks. All experimental feeds were fed to triplicate groups of rockfish twice daily for 8 weeks. Superior weight gain, specific growth rate (SGR), and feed intake of rockfish fed the C20J60 and C20J80 diets compared to rockfish fed the Con and C20J0 diets were observed. However, the feed utilization, biological indices, proximate composition, amino acid profiles, and blood chemistry of the rockfish were not affected by the dietary treatments. The slightly but not significantly higher economic profit index and growth performance were obtained in the C20J60 diet compared to the C20J80 diet. In conclusion, the C20J60 diet was the most recommendable treatment based on the improvement in growth performance (weight gain and SGR) and feed intake of rockfish, and the highest economic return to farmers.

## 1. Introduction

Fish meal (FM) is generally made from wild-caught, small marine pelagic fish and it has been utilized as a key protein source in aquafeeds because of its high protein content, balanced amino acid (AA) profile, and excellent palatability and digestibility [1,2,3]. However, the declining wild fisheries stock commonly used as an FM source and increasing FM price have severely restricted the use of FM in aquafeeds. Therefore, employing an alternative protein source that is not only economically inexpensive but also environmentally friendly for FM in fish feeds will help to minimize FM dependence and improve the economic return for farmers. The alternative sources of FM mainly come from plant protein, animal protein, and single-cell protein sources [4]. Animal protein sources with high protein and lipid content, balanced AA profiles, and excellent protein digestibility have generally been considered more suitable alternative for FM in formulated aquafeeds compared to plant protein sources [4,5].

Chicken by-product, produced from chicken processing plants is an environmentally friendly and inexpensive source of animal protein to replace FM in aquafeeds [6,7]. Chicken by-product contains viscera, heads, bones, blood, and feathers, while viscera and feather account for about 30% and 10% of this waste, respectively [8]. Improper disposal of chicken by-product could cause environmental pollution, diseases, and the loss of useful biological resources, such as protein, lipid, and enzymes [8]. Replacements of FM with chicken by-product meal (CBM), chicken waste meal, chicken intestine, and chicken plasma powder have been reported in the feeds of olive flounder (*Paralichthys olivaceus*) and abalone (*Haliotis discus hannai*) [6,7], Asian sea bass (*Lates calcarifer*) [9], grass carp (*Ctenopharyngodon idella*) [10], and largemouth bass (*Micropterus salmoides*) [11], respectively. In particular, Ha et al. [7] demonstrated that 50% FM replacement with CBM in the 65% FM-based diet of olive flounder could be made without inducing any unfavorable effects on growth and feed availability. Furthermore, up to 10% FM protein in a 35% FM-based diet could be substituted with chicken waste meal without producing any negative impact on the growth and feed availability of Asian seabass [9].

The palatability and attractiveness of feeds are the critical factors to determine the success of feed manufacture as good palatability can promote fish’s ingestion of available nutrients [12]. However, increased replacement levels of FM with the alternatives in aquafeeds commonly leads to deteriorated palatability of feed, and eventually brings about reduced feed consumption and growth [2,5,13]. The manipulation of ingredients with excellent palatability in low-FM feeds is an effective method to address this problem [13]. Some synthetic chemicals, such as betaine, taurine, inosine, nucleotides, and nucleosides are normally considered to improve the palatability of feeds for various fish species [14,15,16]. However, their attractiveness to fish varies greatly depending on fish species, feeding patterns, and the type and dosage of attractants [14,17].

Some feed ingredients in formulated fish feeds, such as tuna viscera hydrolysates and jack mackerel (*Trachurus japonicas*) meal (JMM), could improve feed intake [13,17,18,19]. In particular, Chotikachinda et al. [13] unveiled that the inclusion of 3–4% tuna viscera hydrolysates in the 54% poultry by-product meal-based diet enhanced feed palatability and increased the feed consumption and growth of Asian sea bass. Furthermore, JMM containing AA, inosine-5′-monophosphate (IMP), and nucleotides as palatability enhancers have been explored in some fish species [18,20,21,22]. Takakuwa et al. [23] also found that IMP in the muscle extracts of jack mackerel showed the strongest feeding stimulating response to greater amberjack (*Seriola dumerili*). Furthermore, JMM exhibited the strongest attractiveness to olive flounder among 15 ingredients [18], and manipulation of 5% JMM in extruded pellets produced the greatest attractiveness to olive flounder and led to the highest feed consumption and greatest growth. Choi et al. [24] also demonstrated that incorporation of JMM as the main protein source in the feeds of grower walleye pollock (*Gadus chalcogrammus*) resulted in statistical improvements in growth and daily feed consumption compared to walleye pollock fed diets with anchovy meal or pollock meal as the main protein source. Thus, the application of JMM in low FM diets, commonly resulting in deteriorated feed intake and reduced growth performance, can resolve their disadvantages in fish culture. 

Rockfish is a viviparous marine teleost belonging to the *Sebastidae* family [25] and are widely dispersed over the Northwest Pacific coast [26]. However, the natural stock of rockfish has dramatically decreased in recent years, primarily due to habitat deterioration caused by overfishing and pollution [26]. Meanwhile, rockfish aquaculture industry has rapidly expanded since 1987 [25]. As the second-highest cultured marine fish species, its aquaculture production reached 16,189 metric tons in Korea in 2022 [27]. With the widespread cultivation of rockfish in the Eastern Asia, many nutritionists are paying attention to development of the rockfish feeds [19,22,28,29,30,31,32,33,34]. The growth and feed intake of rockfish improved with increased incorporation levels of JMM from 0% to 40% at the expense of FM in diets, and optimum inclusion levels of JMM in diets were estimated to be 39.7 and 40.3% of FM based on weight gain and feed intake, respectively [19]. The highest feeding attractant response of rockfish was also noticed in JMM among 16 crude protein sources [29]. 

Application of low FM diets in practical feeding is unavoidable for sustainable fish culture [35]. Our earlier study [36] also proved that the replacement of FM with up to 20% CBM could be made without deteriorating the growth and feed intake of rockfish. Based on the substitutability of CBM for FM and the strong attractiveness of JMM to rockfish, this study was designed to elucidate the impact on the growth performance and feed availability of rockfish from including JMM in diets which otherwise substitute 20% FM with CBM. 

## 2. Materials and Methods

### 2.1. Experimental Design 

Fish meal (FM) (anchovy meal; USD 1.84/kg; USD = 1304 KRW) and jack mackerel meal (JMM) (USD 2.57/kg) were imported from Chile, and chicken by-product meal (CBM) was bought from Chamfre Co., Ltd. (Buan-gun, Jeollabuk-do, Republic of Korea) (USD 0.88/kg). Six experimental diets were prepared to be isonitrogenous (50.0%) and isolipidic (15.5%) (Table 1), which met dietary protein and lipid requirements for the growth of rockfish [28,31]. A percentage of 55% FM and 12% fermented soybean meal were contained in the control (Con) diet as the main protein sources. Additionally, 21.5% wheat flour and 4.5% of each fish and soybean oils were included as the carbohydrate and lipid sources, respectively, in the Con diet. A percentage of 20% FM was replaced by CBM in the Con diet, based on Li and Cho’s study [36], and then the graded (0, 20, 40, 60, and 80%) levels of JMM were included at the cost of FM, named as the C20J0, C20J20, C20J40, C20J60, and C20J80 diets, respectively. All the ingredients were well mixed with water at a ratio of 3:1 before being pelletized by a laboratory pellet extruder. After that, the experimental pellets were dried in an electronic dryer at 40 °C for 24 h and then kept at −20 °C until use.

### 2.2. Feeding Conditions

Juvenile rockfish with similar sizes were bought from a local hatchery (Buan-gun, Jeollabuk-do, Republic of Korea) and acclimated to the feeding conditions for 2 weeks before the start of the feeding experiment. During the 2-week acclimation period, all the rockfish were kept in a 1-ton polyvinyl circular tank (water volume: 750 L). The fish were hand-fed a commercial extruded pellet containing 50% crude protein and 13% crude lipid (National Federation of Fisheries Cooperatives Feed, Uiryeong-gun, Gyeongsangnam-do, Republic of Korea) twice a day at a ratio of 2–3% biomass of the fish. Then, 540 juvenile rockfish (initial weight of 11.2 g) were assigned to eighteen 50 L flow-through tanks (30 rockfish/tank). The appropriate aeration and 4.3 L/min of the mixed sand-filtered seawater and underground seawater were continuously supplied. The photoperiod complied with natural circumstances. All fish were hand-fed to satiation twice a day (08:30 and 17:30) for 8 weeks.

During the 8-week feeding experiment, water quality was monitored daily by a multiparameter water quality meter (AZ-8603, AZ Instrument, Taichung, Taiwan). The water temperature changed from 17.2 to 23.1 °C (20.7 ± 1.52 °C; mean ± SD), salinity changed from 30.8 to 32.5 g/L (31.5 ± 0.39 g/L), DO changed from 7.3 to 7.8 mg/L (7.5 ± 0.12 mg/L), and pH changed from 7.4 to 7.7 (7.5 ± 0.07). The tank bottom was cleaned every day after feeding in the morning, and dead fish were immediately removed from the tank upon observation.

### 2.3. Growth and Biological Indices of Rockfish

Upon the completion of the 8-week feeding experiment, all live fish were starved for 24 h, and then anesthetized with tricaine methanesulfonate (MS222) at a concentration of 100 mg/L. The number of surviving rockfish in each tank was counted and total weight was collectively measured. Then, 10 rockfish were randomly chosen to measure their total length and weight individually for the determination of their condition factor (CF). After that, these 10 fish were dissected, and the visceral and liver organs were separated and weighed to evaluate their viscerosomatic index (VSI) and hepatosomatic index (HSI), respectively. The following formulas were used: specific growth rate (SGR, %/day) = [(Ln final weight of rockfish − Ln initial weight of rockfish) × 100]/days of feeding (56 days), feed conversion ratio (FCR) = feed consumption/(final total weight of rockfish + total weight of dead rockfish − initial total weight of rockfish), protein efficiency ratio (PER) = weight gain of rockfish/protein consumption, protein retention (PR, %) = protein gain of rockfish × 100/protein consumption, CF (g/cm^3^) = body weight of rockfish (g) × 100/total length of rockfish (cm)^3^, HSI (%) = liver weight of rockfish × 100/body weight of rockfish, and VSI (%) = viscera weight of rockfish × 100/body weight of rockfish.

### 2.4. Biochemical Analysis of the Experimental Feeds and Rockfish

Ten fish before the feeding trial and six fish from each tank after the completion of the 8-week feeding trial were randomly sampled and homogenized for the biochemical composition of the whole-body rockfish. The chemical composition of the experimental feeds and whole-body rockfish were determined according to the AOAC [37] standard procedure. The AA in the experimental feeds and whole-body rockfish were analyzed by using an AA analyzer (L-8900 Auto-analyzer; Hitachi, Tokyo, Japan), and FA was analyzed by gas chromatography (HP 6890, Hewlett Packard Ltd., Palo Alto, CA, USA) as previously described in Lee et al.’s study [32]. 

### 2.5. Analysis of Plasma and Serum Parameters of Rockfish

To evaluate the plasma and serum measurements of the rockfish, blood was collected via 5 heparinized syringes and 5 syringes from the caudal vein of ten fish per tank, and then centrifuged (2700× *g*) at 4 °C for 10 min. All blood samples were stored at −70 °C until analysis. Plasma parameters were measured by using an automatic analyzer (Fuji Dri-Chem NX500i, Fujifilm, Tokyo, Japan). Serum was used to assess superoxide dismutase (SOD) and lysozyme activity. The SOD was determined by ELISA kit (MyBioSource, cat. No. MBS705758) according to the manufacturer’s instructions. The turbidimetric assay for lysozyme activity was carried out following Lange et al.’s study [38]. The same procedures and methods were used as previously described [39]. 

### 2.6. Economic Analysis of the Experiment

The economic analysis of the feeding experiment was calculated by using the formula reported in Martínez-Llorens et al.’s study [40]: economic conversion ratio (ECR, USD/kg) = feed consumption (kg/fish) × diet price (USD/kg)/weight gain of fish (kg/fish), economic profit index (EPI, USD/fish) = final weight (kg/fish) × fish sale price (USD/kg) − feed consumption (kg/fish) × diet price (USD/kg). The price (USD/kg) of each ingredient was as follows: FM = 1.84, CBM = 0.88, JMM = 2.57, fermented soybean meal = 0.66, wheat flour = 0.52, fish oil = 2.61, soybean oil = 1.69, vitamin mix = 7.82, mineral mix = 6.29, and choline = 1.23. Rockfish sale price was calculated at USD 9.53/kg [27].

### 2.7. Statistical Analysis

After the normality and homogeneity tests, the data were subjected to one-way ANOVA and Tukey’s HSD test in SPSS version 26.0 (SPSS Inc., Chicago, IL, USA). All parameters of the rockfish were subjected to orthogonal polynomial contrast and regression analysis to determine the most suitable models (linear, quadratic, or cubic) between the parameters and JMM inclusion levels in feeds which substitute 20% FM with CBM excluding the Con diet. Percentage data were arcsine-transformed prior to statistical analysis.

## 3. Results

### 3.1. AA and FA Profiles of the Experimental Feeds

All essential AA (EAA) were relatively high in fish meal (FM) over those in chicken by-product meal (CBM) and jack mackerel meal (JMM), except for arginine and histidine (Table 2). The AA profiles in FM, CBM, and JMM were well reflected in those in the experimental feeds. However, with increased incorporation levels of JMM in diets which substitute 20% FM with CBM, arginine and histidine content tended to increase but all other EAA content tended to decrease. The requirements of arginine (2.78% of the diet) [41] and lysine (2.99% of the diet) [42] for growth of the rockfish were fulfilled in all experimental feeds (2.97–3.12% and 3.43–3.73% of diets, respectively). Glutamic acid was the richest non-EAA (NEAA) in all the diets. 

The total content of saturated FA (∑SFA), arachidonic acid (ARA, 20:4n-6), and docosahexanoic acid (DHA, 22:6n-3) were relatively high in FM over those in CBM and JMM, but low for the total content of monounsaturated FA (∑MUFA) (Table 3). The content of eicosapentanoic acid (EPA, 20:5n-3) and total content of n-3 highly unsaturated FA (∑n-3 HUFA) were relatively high in JMM over those in FM and CBM. However, the ∑MUFA in CBM was relatively higher than that in FM and JMM. The FA profiles in FM, CBM, and JMM were well reflected in the experimental feeds. With the elevated incorporation levels of JMM in diets replacing 20% FM with CBM, the ∑SFA and DHA decreased but the ∑MUFA, EPA, and ∑n-3 HUFA increased. The requirement of ∑n-3 HUFA (5.88% of total FA) [30] for growth of the rockfish was fulfilled in all experimental feeds (6.68–7.47% of total FA).

### 3.2. Survival, Growth Performance, Feed Availability, and Biometric Indices of Fish

**Figure 1:**
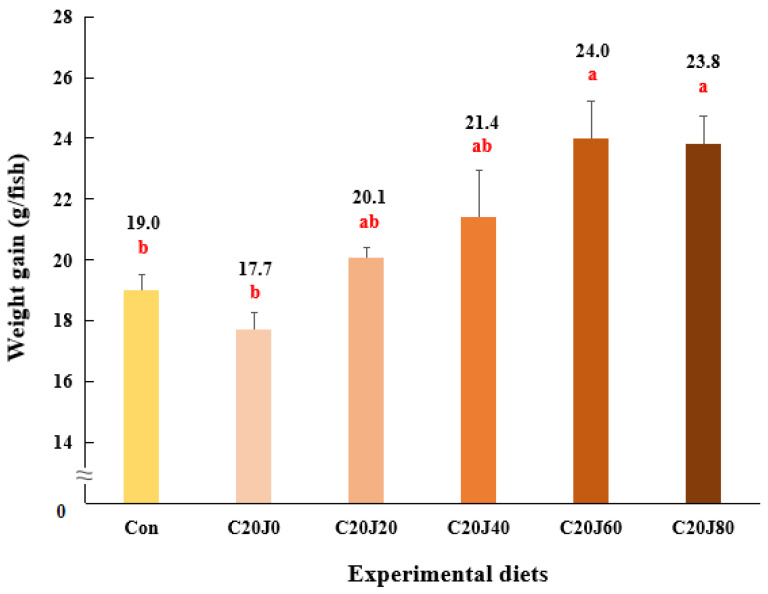
Weight gain (g/fish) of rockfish (*Sebastes schlegeli*) fed the experimental diets for 8 weeks (mean of triplicate ± SE) (*p* < 0.003). Orthogonal polynomial contrast (linear, *p* = 0.001; quadratic, *P* = 0.328; cubic, *p* = 0.604) and the best-fitting model showed a linear (Y = 0.0793X + 18.2467, *p* < 0.001, adjusted R^2^ = 0.6435) relationship between the weight gain of rockfish and dietary inclusion levels of jack mackerel meal (JMM). The different letters under numerical values indicate significant differences among dietary treatments.

**Figure 2:**
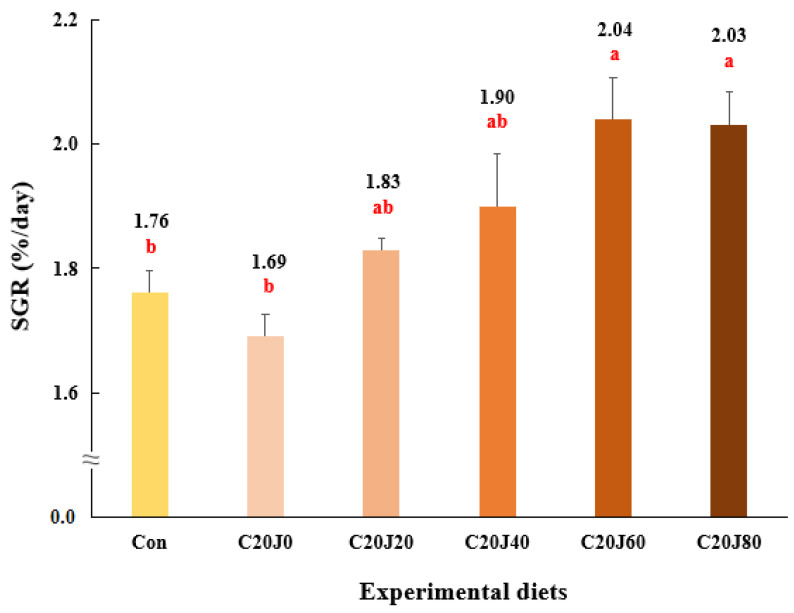
Specific growth rate (SGR, %/day) of rockfish (*Sebastes schlegeli*) fed the experimental diets for 8 weeks (mean of triplicate ± SE) (*p* < 0.003). Orthogonal polynomial contrast (linear, *p* = 0.001; quadratic, *p* = 0.290; cubic, *p* = 0.678) and the best-fitting model showed a linear (Y = 0.0043X + 1.7256, *p* < 0.001, adjusted R^2^ = 0.6391) relationship between the SGR of rockfish and dietary inclusion levels of jack mackerel meal (JMM). SGR (%/day) = [(Ln final weight of rockfish − Ln initial weight of rockfish) × 100]/days of feeding. The different letters under numerical values indicate significant differences among dietary treatments.

The survival of rockfish (91.1–98.9%) was not significantly (*p* > 0.2) changed by the dietary treatments (Table 4). Weight gain and SGR of rockfish fed the C20J60 and C20J80 feeds were significantly (*p* < 0.003 for both) greater than those of the rockfish fed the Con and C20J0 feeds but were not significantly different from those of rockfish fed the C20J20 and C20J40 diets (Figure 1 and Figure 2, respectively). In terms of the orthogonal contrast, weight gain and SGR of rockfish, these exhibited significant (*p* = 0.001 for both) linear relationships with the incorporated levels of JMM in the diets which substitute 20% FM with CBM. The most appropriate linear models were observed between dietary inclusion levels of JMM and weight gain (Y = 0.0793X + 18.2467, *p* < 0.001, adjusted R^2^ = 0.6435) and SGR (Y = 0.0043X + 1.7256, *p* < 0.001, adjusted R^2^ = 0.6391) of rockfish. 

**Figure 3:**
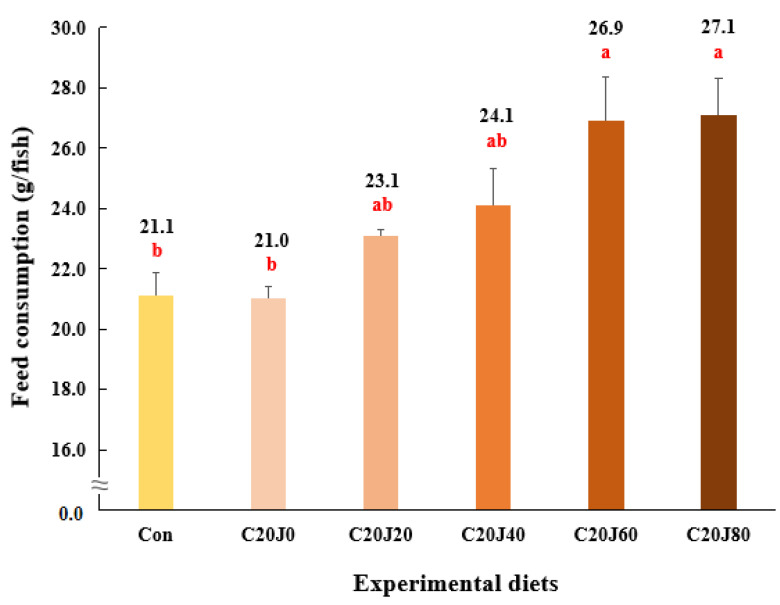
Feed consumption (g/fish) of rockfish (*Sebastes schlegeli*) fed the experimental diets for 8 weeks (mean of triplicate ± SE) (*p* < 0.03). Orthogonal polynomial contrast (linear, *p* = 0.001; quadratic, *p* = 0.627; cubic, *p* = 0.641) and the best-fitting model showed a linear (Y = 0.0793X + 21.2533, *p* < 0.001, adjusted R^2^ = 0.6551) relationship between feed consumption of rockfish and dietary inclusion levels of jack mackerel meal (JMM). The different letters under numerical values indicate significant differences among dietary treatments.

**Figure 4:**
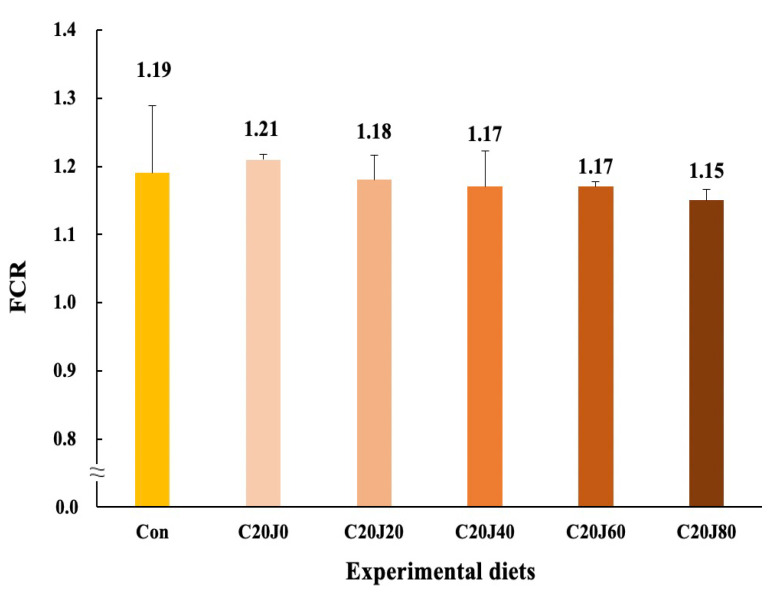
Feed conversion ratio (FCR) of rockfish (*Sebastes schlegeli*) fed the experimental diets for 8 weeks (mean of triplicate ± SE) (*p* > 0.9). Orthogonal polynomial contrast (linear, *p* = 0.158; quadratic, *p* = 0.823; cubic, *p* = 0.669) did not show any relationship between FCR of rockfish and dietary inclusion levels of jack mackerel meal (JMM). FCR = Feed consumption/(final total weight of rockfish + total weight of dead rockfish − initial total weight of rockfish).

The rockfish fed the C20J60 and C20J80 feeds exhibited superior (*p* < 0.03) feed consumption compared to the rockfish fed the Con and C20J0 feeds but were not statistically (*p* > 0.05) different from that of rockfish fed the C20J20 and C20J40 feeds (Figure 3). In regard to the orthogonal contrast, feed consumption of rockfish showed a significant (*p* = 0.001) linear relationship with incorporation levels of JMM in diets which substitute 20% FM with CBM. The most appropriate linear model was observed between dietary inclusion levels of JMM and feed consumption (Y = 0.0793X + 21.2533, *p* < 0.001, adjusted R^2^ = 0.6551). However, the FCR (1.15–1.21) of rockfish was not significantly (*p* > 0.9) affected by the dietary treatments (Figure 4). Similarly, the PER (1.69–1.79) and PR (28.3–30.4%) of the rockfish were not significantly (*p* > 0.8 and *p* > 0.6, respectively) changed by the dietary treatments.

None of the CF, HSI, and VSI of fish were significantly (*p* > 0.3, *p* > 0.8, and *p* > 0.4, respectively) impacted by the dietary treatments.

### 3.3. Biochemical Composition of the Whole-body Rockfish

The moisture (70.0–70.4%), crude protein (16.4–16.7%), crude lipid (7.9–8.1%), and ash (4.0–4.1%) content of the whole-body fish were not significantly (*p* > 0.9, *p* > 0.2, *p* > 0.9, and *p* > 0.8, respectively) altered by the dietary treatments (Table 5).

The AA profiles of the whole-body rockfish were not significantly (*p* > 0.05 for all) changed by the dietary treatments (Table 6). 

The ∑SFA, ∑n-3 HUFA, and DHA content in the whole-body rockfish fed the Con diet were statistically (*p* < 0.001 for all) higher than those in the rockfish fed all the other diets (Table 7). However, the ∑MUFA and EPA content in the whole-body rockfish fed the C20J80 diet were statistically (*p* < 0.001 for both) higher than those in the rockfish fed all the other diets, except for the rockfish fed the C20J60 diet. In regard to the orthogonal contrast, the ∑SFA and ∑n-3 HUFA in the whole-body rockfish showed significant (*p* = 0.001 and *p* = 0.033, respectively) linear relationships with dietary incorporation levels of JMM, while the ∑MUFA exhibited significant linear (*p* = 0.002) and quadratic (*p* = 0.008) relationships. The most appropriate models showed linear (*p* < 0.001, adjusted R^2^ = 0.920), quadratic (*p* < 0.001, adjusted R^2^ = 0.864), and linear (*p* < 0.03, adjusted R^2^ = 0.294) relationships between dietary incorporation levels of JMM and the ∑SFA, ∑MUFA, and ∑n-3 HUFA in the whole-body fish, respectively. Furthermore, the most appropriate models showed linear relationships between the dietary incorporation levels of JMM and EPA (*p* < 0.001, adjusted R^2^ = 0.916) and DHA (*p* < 0.001, adjusted R^2^ = 0.086) in the whole-body fish. 

### 3.4. Plasma and Serum Chemistry of Rockfish

Plasma aspartate aminotransferase (AST) (79.8–84.6 U/L), alanine aminotransferase (ALT) (26.1–30.4 U/L), alkaline phosphatase (ALP) (139.1–191.6 U/L), total bilirubin (T-BIL) (0.4–0.6 mg/dL), total cholesterol (T-CHO) (222.9–246.7 mg/dL), triglyceride (TG) (442.4–453.3 mg/dL), total protein (TP) (3.9–4.2 g/dL), and albumin (ALB) (0.8–1.0 g/dL) were not significantly (*p* > 0.05 for all) altered by the dietary treatments (Table 8).

Serum SOD (3.8–4.4 ng/mL) and lysozyme activity (189.7–301.1 U/mL) were not significantly (*p* > 0.9 and *p* > 0.6, respectively) influenced by the dietary treatments.

### 3.5. Economic Analysis of the Present Study

The price of the diets which substitute 20% FM with CBM increased with elevated incorporation levels of JMM (Table 9). The ECR of the Con, C20J0, and C20J20 diets were significantly (*p* < 0.01) lower than that of the C20J80 diet, but not significantly (*p* > 0.05) different from that of the C20J40 and C20J60 diets. The EPI of the C20J60 and C20J80 diets were significantly (*p* < 0.02) superior to the C20J0 diet, but comparable the Con, C20J20, and C20J40 diets. In terms of orthogonal contrast, the ECR and EPI exhibited significant (*p* = 0.001 and *p* = 0.002, respectively) linear relationships with dietary incorporation levels of JMM. The most appropriate models showed linear relationships between dietary incorporation levels of JMM and ECR (*p* < 0.001, adjusted R^2^ = 0.796), and EPI (*p* < 0.001, adjusted R^2^ = 0.551). 

## 4. Discussion

Our earlier study [36] found that up to 20% of fish meal (FM) could be replaceable by chicken by-product meal (CBM) without deteriorating growth and feed utilization of rockfish when juvenile rockfish were supplied with a 55% FM-based feed or one of the feeds which replace 10, 20, 30, 40, and 50% FM with CBM. Kim et al. [29] unveiled that jack mackerel meal (JMM) showed the strongest attractiveness to rockfish among 16 crude protein sources. That is why, in this study, we included the graded levels (0–80%) of JMM in low-FM diets which replace 20% FM with CBM. That is also why we tried to figure out the impact on growth and feed availability of rockfish from manipulating the graded levels (0–80%) of JMM in low-FM diets which replace 20% FM with CBM. No statistical differences in weight gain, SGR, and feed intake of the rockfish fed the Con and C20J0 diets supported our early finding [36]. 

Linear improvement relationships in weight gain, SGR and feed intake of rockfish vs. incorporated JMM levels in low-FM diets which substitute 20% FM with CBM in this experiment indicated that JMM effectively improved feed intake of rockfish which were fed the low FM diets, eventually resulting in improved growth performance. However, no statistical differences in growth, SGR, and feed intake of rockfish which were fed the C20J60 and C20J80 diets implied that the C20J60 diet appeared to be the most recommended dietary treatment in this experiment. However, incorporated JMM levels up to 40% in the 55% FM-based diet improved weight gain, SGR, and feed intake of rockfish; however, JMM inclusion levels higher than 40% (60% and 100%) did not improve further when juvenile rockfish were fed with a 55% FM-based diet or one of the diets which include 1, 3, 5, 10, 20, 40, 60, and 100% JMM at the cost of FM in the 56-day feeding experiment [19]. They also concluded that optimum inclusion levels of JMM were estimated to be 39.7 and 40.3% based on weight gain and feed consumption, respectively. Similarly, rockfish fed an extruded pellet incorporating 5% JMM as a feed enhancer instead of anchovy meal brought out the best weight gain and highest feed intake [22]. 

Feed ingredients from marine animal origins, such as FM, fish hydrolysates, fish oil, shrimp meal, and krill meal in diets are commonly known to have positive palatability for several fish species [43]. Wei et al. [44] revealed that substituting up to 75% FM with Antarctic krill (*Euphausia superba*) meal in diets could be made without inhibiting growth performance when large yellow croaker (*Larimichthys crocea*) was provided with a 40% FM-based diet or one of the diets which replace 15, 30, 45, 60, and 75% FM with Antarctic meal for 9 weeks. They also stressed that dietary FM replacement with Antarctic meal resulted in improved feed intake and good skin color in muscle of large yellow croakers, and they explained that the improved feed intake might have been due to the excellent palatability of krill meal or leaching of smell-releasing chemical compounds from krill meal into the water, which stimulated feed searching behavior in fish. Similarly, the tissue of jack mackerel contains feeding attractants, such as AA, IMP, and nucleotides [21], which might trigger the ingestion of rockfish fed the C20J60 and C20J80 diets, resulting in improved growth performance in this experiment. Furthermore, IMP derived from mackerel muscle extracts promoted feeding activity of the yellowtail (*Seriola quinqueradiata*) [20], Pacific bluefin tuna (*Trachrus japonicus*) [45], and greater amberjack (*Seriola dumerili*) [23]. Ikeda et al. [46] also found that among the components of the synthetic muscle extracts of jack mackerel, AA, especially histidine, exhibited the highest feeding stimulant activity to olive flounder. Likewise, Kim and Cho [22] revealed that AA, such as alanine, glycine, and histidine, and free histidine content in diets, showed correlations with the growth performance of rockfish. Therefore, increased alanine, glycine, and, in particular, histidine content in diets which replace 20% FM with CBM with incorporated levels of JMM could improve the palatability and eventually the feed intake and growth of rockfish in this experiment.

Except for arginine, all EAA content in CBM were relatively lower than those in FM. However, all EAA profiles of JMM were similar to those of FM (anchovy meal), except for the high content of histidine and the low content of leucine and tryptophan in the former. Several factors including feed intake, water temperature, fish size (age), and fish sex may influence the requirements of AA in diets [47]. Nevertheless, the content of arginine (2.97–3.12% of the diet) and lysine (3.43–3.73% of the diet) in all the experimental feeds fulfilled the requirements (2.78 and 2.99% of the diet, respectively) for rockfish [41,42]. Furthermore, marine fish species require EPA, DHA, and ARA for their appropriate growth and development since they are likely to lack the ability to synthesize long-chain polyunsaturated FA (PUFA) from their 18-carbon precursor FA [48]. The requirement of ∑n-3 HUFA (5.88% of total FA) for rockfish [30] was also met in all the experimental feeds (6.68–7.51% of total FA). 

No remarkable differences were found in the FCR, PER, and PR of rockfish in the present experiment, perhaps demonstrating that the elevated growth performance of rockfish was the result of the improved feed intake. Additionally, the biometric indices of the rockfish were not changed by the dietary treatments, consistent with the result of our previous study [19] showing that the inclusion of JMM in rockfish feeds did not influence feed utilization and biometric indices. Similarly, feed utilization and biometric indices of olive flounder were not changed when 25 and 50% of FM were substituted with various animal proteins (tuna by-product meal, CBM, and meat meal) and 12% JMM inclusion as feed stimulants [49]. Additionally, the FCR of Atlantic salmon (*Salmo salar*) and Atlantic halibut (*Hippoglossus hippoglossus*) were not influenced when fish were fed with a 61% FM-based diets or one of the diets which incorporate 20, 40, and 60% of krill meal or 40% amphipods meal at the expense of FM [50].

No remarkable differences in the proximate composition and AA profiles of rockfish were found in this experiment, consistent with other studies in which incorporation of JMM in feeds did not affect the proximate composition and AA profiles of whole-body rockfish [19] and olive flounder [17], and the chemical composition of the muscle and liver of walleye pollock [24]. Similarly, the incorporation of feed ingredients in diets did not alter the whole-body chemical composition and AA profiles of the large yellow croaker [44], and chemical composition of the red sea bream (*Pagrus major*) [51,52], olive flounder [53], and Atlantic salmon [50]. 

The n-3 HUFA are non-dispensable FAs for all vertebrates including fish and humans [54]. Like all other vertebrates, fish require n-3 HUFA for normal growth and development including reproduction to maintain the normal structure and function of cell membranes [55]. The low content of ∑n-3 HUFA in diets might lead to a decrease in the content of these non-dispensable FAs in fish, which could have adverse impact on consumer health [56]. Because of the negligible amount of EPA, DHA, and ∑n-3 HUFA in CBM, the C20J0 diet exhibited the lowest ∑n-3 HUFA content. However, elevated JMM inclusion levels in low FM diets substituting 20% FM by CBM resulted to decreased ∑SFA and ∑n-3 HUFA, but increased ∑MUFA, eventually bringing about decreased ∑SFA and ∑n-3 HUFA, but increased ∑MUFA of the whole-body rockfish at the end of the 8-week feeding experiment. Likewise, FA profiles of the whole-body fish were well reflected from dietary FA profiles [2,48,57]. The dorsal muscle FA profiles of large yellow croakers were also influenced by FM substitution with krill meal in diets [44]. Suontama et al. [50] demonstrated that the EPA content of the muscle of Atlantic salmon was changed by incorporating krill meal and amphipods meal in diets, while the ∑SFA, ∑MUFA, and total content of PUFA were unaffected by dietary treatments. Unlike this experiment, the FA profiles of olive flounder were not influenced by dietary treatments when 12% JMM was included in diets which replace 25 and 50% FM with animal proteins [49]. 

In numerous pathological and ecotoxicological studies, plasma and serum parameters have been employed as the typical indicators of fish health status [32]. AST and ALT can be detected in the blood, liver, and spleen of fish. When fish are healthy, they maintain modest activity; however, when there is tissue necrosis or disease, they are released, resulting in higher activity [58]. In addition, SOD and lysozyme activity are reliable indicators for determining the effect of nutrition on the health status of fish [59]. However, none of the plasma and serum measurements of rockfish were changed by the dietary treatments in this experiment, agreeing with our early study [49] showing that the incorporation of JMM in low-FM diets which replace 25 and 50% FM with various animal proteins did not influence the plasma and serum parameters of olive flounder. However, the serum T-BIL, TP, and lysozyme activity of the Asian sea bass were changed when Asian sea bass were fed with the 72.6% FM-based diet or one of the diets which replace 80, 85, and 90% FM with poultry by-product meal with supplementation of 3.5–7% tuna hydrolysate and 5–10% black soldier fly (*Hermetia illucens*) larvae meal [60]. Additionally, the plasma ALT and AST of Atlantic salmon were altered when fish were provided with diets incorporating krill meal or amphipod meal [50].

In regard to the economic analysis of this study, the C20J60 diet produced a slightly but not significantly higher EPI when compared to the C20J80 diet. This also well supported by the growth of the rockfish fed the C20J60 diet. Thus, when compared with the previous studies, the results obtained in this innovative study suggested that the manipulation of JMM in the low-FM diets is a good strategy to improve the palatability of diets and feed consumption for rockfish. Furthermore, the C20J60 diet appears to be the most recommendable dietary treatment as farmers can receive a high economic return in practical aquaculture.

## 5. Conclusions

The manipulation of jack mackerel meal in rockfish diets by replacing 20% fish meal with chicken by-product meal effectively improved growth performance and feed intake, but did not change feed utilization, biological indices, chemical composition, AA profiles, and serum chemistry of rockfish. Furthermore, the C20J60 diet is the most recommended based on improvement in the growth and feed intake of rockfish and high economic return to farmers.

## Figures and Tables

**Table 1:** Ingredient and chemical composition of the experimental diets (%, dry matter basis).

	Experimental Diets					
	Con	C20J0	C20J20	C20J40	C20J60	C20J80
Ingredient (%, DM)						
Fish meal (FM) ^1^	55.0	44.0	33.0	22.0	11.0	0.0
Chicken by-product meal (CBM) ^2^		12.6	12.6	12.6	12.6	12.6
Jack mackerel meal (JMM) ^3^			11.0	22.4	33.8	45.0
Fermented soybean meal	12.0	12.0	12.0	12.0	12.0	12.0
Wheat flour	21.5	20.9	20.8	20.3	19.9	19.6
Fish oil	4.5	4.5	4.5	4.5	4.5	4.5
Soybean oil	4.5	3.5	3.6	3.7	3.7	3.8
Mineral mix ^4^	1.0	1.0	1.0	1.0	1.0	1.0
Vitamin mix ^5^	1.0	1.0	1.0	1.0	1.0	1.0
Choline	0.5	0.5	0.5	0.5	0.5	0.5
Nutrients (%, DM)						
Dry matter	94.4	94.3	94.1	93.9	94.6	94.3
Crude protein	50.4	50.0	50.1	50.4	50.3	50.6
Crude lipid	15.5	14.4	14.7	15.6	15.7	15.7
Ash	9.1	9.1	9.5	9.4	9.5	9.9

^1^ Fish meal (FM, anchovy meal) (crude protein: 73.4%, crude lipid: 10.7%, ash: 14.0%) imported from Chile was purchased from KOFEC Co., Ltd. (Naju, Jeollanam-do, Republic of Korea) (USD 1.84/kg FM, USD = 1304 KRW). ^2^ Chicken by-product meal (CBM) (crude protein: 65.4%, crude lipid: 17.6%, ash: 8.5%) was purchased from Chamfre Co., Ltd. (Buan-gun, Jeollabuk-do, Republic of Korea) (USD 0.88/kg CBM). ^3^ Jack mackerel meal (JMM) (crude protein: 72.2%, crude lipid: 9.9%, ash: 14.3%) imported from Chile was purchased from Daekyung Oil & Transportation Co., Ltd. (Busan, Republic of Korea) (USD 2.57/kg JMM). ^4^ Mineral mix contained the following ingredients (g/kg mix): MgSO_4_·7H_2_O, 80.0; NaH_2_PO_4_·2H_2_O, 370.0; KCl, 130.0; ferric citrate, 40.0; ZnSO_4_·7H_2_O, 20.0; Ca-lactate, 356.5; CuCl, 0.2; AlCl_3_·6H_2_O, 0.15; KI, 0.15; Na_2_Se_2_O_3_, 0.01; MnSO_4_·H_2_O, 2.0; CoCl_2_·6H_2_O, 1.0. ^5^ Vitamin mix contained the following amount which were diluted in cellulose (g/kg mix): L-ascorbic acid, 121.2; DL-α-tocopheryl acetate, 18.8; thiamin hydrochloride, 2.7; riboflavin, 9.1; pyridoxine hydrochloride, 1.8; niacin, 36.4; Ca-D-pantothenate, 12.7; myo-inositol, 181.8; D-biotin, 0.27; folic acid, 0.68; p-aminobenzoic acid, 18.2; menadione, 1.8; retinyl acetate, 0.73; cholecalciferol, 0.003; cyanocobalamin, 0.003.

**Table 2:** Amino acid (AA) profiles (% of the diet) of the main feed ingredients (FM, CBM, and JMM) and experimental diets.

	Ingredients			Requirement	Experimental Diets					
	FM	CBM	JMM		Con	C20J0	C20J20	C20J40	C20J60	C20J80
Essential amino acid (%)										
Arginine	3.87	3.96	3.90	2.78 ^1^	2.97	3.07	3.10	3.10	3.11	3.12
Histidine	1.89	1.34	2.83		1.41	1.30	1.45	1.63	1.72	1.80
Isoleucine	2.89	2.43	2.76		2.19	2.10	2.07	2.04	2.01	1.99
Leucine	5.03	4.47	4.83		3.87	3.69	3.61	3.53	3.47	3.43
Lysine	5.36	4.20	5.31	2.99 ^2^	3.73	3.62	3.58	3.52	3.49	3.43
Phenylalanine	2.64	2.46	2.60		2.14	2.03	2.00	1.97	1.95	1.90
Threonine	2.95	2.53	2.88		2.25	2.17	2.12	2.09	2.07	2.02
Tryptophan	0.69	0.56	0.57		0.27	0.24	0.22	0.21	0.18	0.16
Valine	3.34	2.99	3.22		2.46	2.32	2.29	2.26	2.24	2.20
∑EAA ^3^	28.66	24.94	28.90		21.29	20.54	20.44	20.35	20.24	20.05
Non-essential amino acid (%)										
Alanine	4.16	4.00	4.29		2.99	2.90	2.93	3.00	3.07	3.13
Aspartic acid	6.07	5.00	5.91		4.75	4.63	4.55	4.51	4.46	4.42
Glutamic acid	8.34	8.03	8.34		7.28	7.02	7.07	7.05	7.06	7.08
Glycine	3.76	4.99	4.29		2.78	3.27	3.44	3.60	3.77	3.89
Proline	2.74	3.63	2.98		2.49	2.63	2.70	2.79	2.85	2.93
Serine	2.58	2.46	2.59		2.12	2.09	2.13	2.17	2.19	2.23
Tyrosine	1.82	1.53	1.71		1.33	1.26	1.23	1.20	1.17	1.16
∑NEAA ^4^	29.47	29.64	30.11		23.74	23.80	24.05	24.32	24.57	24.84

^1^ Data were obtained in [41]. ^2^ Data were obtained in [42]. ^3^ ∑EAA: total content of essential amino acids. ^4^ ∑NEAA: total content of non-essential amino acids.

**Table 3:** Fatty acid (FA) profiles (% of total FA) of main feed ingredients (FM, CBM, and JMM) and experimental diets.

	Ingredients				Experimental Diets					
	FM	CBM	JMM	Requirement	Con	C20J0	C20J20	C20J40	C20J60	C20J80
C14:0	4.20	1.78	4.29		1.91	1.48	1.52	1.60	1.65	1.78
C16:0	23.87	26.18	21.26		15.94	16.13	15.84	15.66	15.51	15.30
C18:0	8.05	8.52	7.58		4.96	5.23	5.14	5.06	4.97	4.91
C20:0	0.10	0.16	0.10		0.20	0.24	0.22	0.22	0.21	0.21
C22:0	0.30	0.17	0.16		0.36	0.29	0.28	0.26	0.26	0.25
C24:0	0.68	0.28	0.50		0.56	0.43	0.40	0.39	0.37	0.37
∑SFA ^1^	37.20	37.09	33.89		23.93	23.80	23.40	23.19	22.97	22.82
C16:1n-7	5.62	7.27	6.84		3.15	3.43	3.56	3.68	3.91	3.96
C18:1n-9	23.30	40.51	23.21		31.89	33.48	33.43	33.40	33.35	33.31
C20:1n-9	1.01	0.51	1.54		1.15	0.94	1.01	1.08	1.15	1.20
C22:1n-9	0.19	0.34	0.16		0.41	0.52	0.48	0.46	0.44	0.43
C24:1n-9	2.69	0.11	4.00		1.20	0.91	1.00	1.11	1.24	1.36
∑MUFA ^2^	32.81	48.74	35.75		37.80	39.28	39.48	39.73	40.09	40.26
C18:2n-6	1.89	11.28	1.33		23.05	24.72	24.40	23.88	23.37	23.10
C18:3n-3	0.70	0.64	0.51		3.47	3.17	3.08	3.03	2.90	2.63
C20:4n-6	2.44	1.38	1.74		0.98	0.79	0.75	0.70	0.68	0.67
C20:5n-3	7.06	0.04	10.98		2.79	2.37	2.56	3.02	3.36	3.56
C22:2n-6	0.60	0.04	0.70		0.37	0.31	0.31	0.33	0.33	0.34
C22:6n-3	14.71	0.13	12.18		4.68	4.31	4.22	4.00	3.93	3.85
∑n-3 HUFA ^3^	21.77	0.17	23.16	5.88 ^4^	7.47	6.68	6.78	7.02	7.29	7.41
Unknown	2.59	0.66	2.92		2.93	1.25	1.80	2.12	2.37	2.77

^1^ ∑SFA: total content of saturated fatty acids. ^2^ ∑MUFA: total content of monounsaturated fatty acids. ^3^ ∑n-3 HUFA: total content of n-3 highly unsaturated fatty acids. ^4^ Data were obtained in [30].

**Table 4:** Survival (%), protein efficiency ratio (PER), protein retention (PR), condition factor (CF), hepatosomatic index (HSI), and visceralsomatic index (VSI) of rockfish fed the experimental diets for 8 weeks.

	Experimental Diets								Orthogonal Contrast			Regression		
	Con	C20J0	C20J20	C20J40	C20J60	C20J80	SEM	*p*-Value	Linear	Quadratic	Cubic	Model	*p-*Value	Adj. R^2^
Initial weight (g/fish)	11.3	11.2	11.2	11.2	11.3	11.3	0.01	-	-	-	-	-	-	-
Final weight (g/fish)	30.3	28.9	31.3	32.7	35.3	35.0	0.65	-	-	-	-	-	-	-
Survival (%)	91.1	96.7	95.6	94.4	92.2	98.9	1.02	>0.2	0.872	0.105	0.223	NR	-	-
PER ^1^	1.79	1.69	1.74	1.76	1.78	1.73	0.02	>0.8	0.274	0.153	0.859	NR	-	-
PR (%) ^2^	30.4	28.3	29.2	29.1	29.3	28.6	0.32	>0.6	0.802	0.520	0.078	NR	-	-
CF (g/cm^3^) ^3^	1.65	1.53	1.53	1.67	1.68	1.64	0.03	>0.3	0.102	0.397	0.328	NR	-	-
HSI (%) ^4^	3.23	3.16	3.13	3.14	2.96	3.05	0.05	>0.8	0.475	0.946	0.689	NR	-	-
VSI (%) ^5^	10.36	9.91	10.45	10.59	10.58	10.44	0.10	>0.4	0.168	0.135	0.731	NR	-	-

Abbreviations: SEM, pooled standard error of treatment means; Adj. R^2^, adjusted R square; NR, no relationship. ^1^ Protein efficiency ratio (PER) = Weight gain of rockfish/protein consumption. ^2^ Protein retention (PR, %) = Protein gain of rockfish × 100/protein consumption. ^3^ Condition factor (CF, g/cm^3^) = Body weight of rockfish (g) × 100/total length of rockfish (cm)^3^. ^4^ Hepatosomatic index (HSI, %) = Liver weight of rockfish × 100/body weight of rockfish. ^5^ Visceralsomatic index (VSI, %) = Viscera weight of rockfish × 100/body weight of rockfish.

**Table 5:** Proximate composition (% of wet weight) of rockfish fed the experimental diets for 8 weeks.

	Experimental Diets								Orthogonal Contrast			Regression		
	Con	C20J0	C20J20	C20J40	C20J60	C20J80	SEM	*p*-Value	Linear	Quadratic	Cubic	Model	*p-*Value	Adj. R^2^
Moisture	70.4	70.2	70.0	70.1	70.4	70.0	0.12	>0.9	0.964	0.913	0.472	NR	-	-
Crude protein	16.7	16.6	16.6	16.5	16.4	16.4	0.04	>0.2	0.154	0.839	0.517	NR	-	-
Crude lipid	8.0	8.0	8.0	8.1	7.9	7.9	0.04	>0.9	0.756	0.493	0.955	NR	-	-
Ash	4.0	4.0	4.0	4.0	4.0	4.1	0.03	>0.8	0.303	0.489	0.858	NR	-	-

Abbreviations: SEM, pooled standard error of treatment means; Adj. R^2^, adjusted R square; NR, no relationship.

**Table 6:** AA profiles (% of wet weight) of the whole-body rockfish fed the experimental diets for 8 weeks.

	Experimental Diets								Orthogonal Contrast		Regression		
	Con	C20J0	C20J20	C20J40	C20J60	C20J80	SEM	*p*-Value	Linear	Quadratic	Model	*p-*Value	Adj. R^2^
Essential amino acid (%)													
Arginine	1.00	0.97	0.95	0.89	0.90	0.95	0.02	>0.3	0.465	0.181	NR	-	-
Histidine	0.35	0.30	0.32	0.32	0.33	0.32	0.01	>0.6	0.404	0.610	NR	-	-
Isoleucine	0.69	0.65	0.68	0.67	0.68	0.67	0.01	>0.8	0.475	0.391	NR	-	-
Leucine	1.08	1.04	1.04	1.06	1.05	1.07	0.01	>0.6	0.211	0.895	NR	-	-
Lysine	1.20	1.17	1.17	1.18	1.17	1.19	0.01	>0.8	0.570	0.847	NR	-	-
Phenylalanine	0.58	0.52	0.54	0.57	0.55	0.56	0.01	>0.3	0.111	0.287	NR	-	-
Threonine	0.70	0.62	0.66	0.67	0.65	0.67	0.01	>0.2	0.125	0.297	NR	-	-
Tryptophan	0.09	0.09	0.08	0.08	0.08	0.08	0.01	>0.9	0.531	0.430	NR	-	-
Valine	0.69	0.62	0.61	0.64	0.64	0.65	0.01	>0.1	0.154	0.928	NR	-	-
Non-essential amino acid (%)													
Alanine	1.07	1.05	1.06	1.06	1.06	1.07	0.01	>0.7	0.121	0.519	NR	-	-
Aspartic acid	1.56	1.51	1.47	1.47	1.48	1.48	0.02	>0.4	0.675	0.469	NR	-	-
Glutamic acid	2.05	2.01	1.93	1.96	1.99	2.03	0.02	>0.1	0.365	0.063	NR	-	-
Glycine	1.25	1.28	1.30	1.31	1.33	1.31	0.01	>0.2	0.306	0.345	NR	-	-
Proline	0.75	0.72	0.71	0.72	0.75	0.72	0.01	>0.8	0.719	0.855	NR	-	-
Serine	0.69	0.68	0.70	0.71	0.72	0.73	0.01	>0.6	0.118	0.661	NR	-	-
Tyrosine	0.42	0.37	0.41	0.40	0.40	0.41	0.01	>0.7	0.301	0.426	NR	-	-

Abbreviations: SEM, pooled standard error of treatment means; Adj. R^2^, adjusted R square; NR, no relationship.

**Table 7:** FA profiles (% of total FA) of the whole-body rockfish fed the experimental diets for 8 weeks.

	Experimental Diets								Orthogonal Contrast			Regression		
	Con	C20J0	C20J20	C20J40	C20J60	C20J80	SEM	*p*-Value	Linear	Quadratic	Cubic	Model	*p-*Value	Adj. R^2^
C14:0	2.35 ^a^	2.31 ^ab^	2.21 ^bc^	2.21 ^bc^	2.19 ^c^	2.15 ^c^	0.02	<0.001	0.002	0.367	0.170	L	<0.002	0.530
C16:0	14.95 ^a^	14.69 ^b^	14.51 ^c^	14.44 ^c^	14.28 ^d^	14.08 ^e^	0.07	<0.001	0.001	0.274	0.146	L	<0.001	0.932
C18:0	4.44 ^bc^	4.41 ^c^	4.53 ^abc^	4.51 ^abc^	4.57 ^a^	4.54 ^ab^	0.02	<0.01	0.006	0.041	0.503	Q	<0.001	0.491
C20:0	0.16	0.18	0.16	0.15	0.16	0.18	0.01	>0.4	0.711	0.048	0.853	NR	-	-
C22:0	0.42 ^a^	0.39 ^ab^	0.37 ^ab^	0.38 ^ab^	0.35 ^b^	0.37 ^ab^	0.01	<0.04	0.193	0.449	0.651	NR	-	-
C24:0	0.52	0.49	0.49	0.49	0.49	0.50	0.01	>0.5	0.734	0.954	0.999	NR	-	-
∑SFA ^1^	22.85 ^a^	22.48 ^b^	22.28 ^c^	22.20 ^cd^	22.05 ^d^	21.82 ^e^	0.08	<0.001	0.001	0.361	0.123	L	<0.001	0.920
C16:1n-7	4.27 ^b^	4.35 ^b^	4.68 ^a^	4.83 ^a^	4.91 ^a^	4.86 ^a^	0.06	<0.001	0.001	0.007	0.743	Q	<0.001	0.796
C18:1n-9	31.87 ^c^	33.23 ^b^	35.42 ^a^	35.43 ^a^	35.83 ^a^	36.01 ^a^	0.38	<0.001	0.001	0.001	0.006	C	<0.001	0.888
C20:1n-9	1.80 ^a^	1.73 ^ab^	1.57 ^bc^	1.61 ^bc^	1.52 ^c^	1.64 ^abc^	0.03	<0.002	0.102	0.019	0.959	Q	<0.03	0.351
C22:1n-9	0.25	0.27	0.26	0.28	0.28	0.27	0.01	>0.6	0.742	0.781	0.514	NR	-	-
C24:1n-9	1.49 ^a^	1.40 ^ab^	1.24 ^cd^	1.17 ^d^	1.35 ^bc^	1.40 ^ab^	0.03	<0.001	0.223	0.001	0.026	C	<0.001	0.714
∑MUFA ^2^	39.69 ^d^	40.07 ^c^	43.18 ^b^	43.33 ^b^	43.89 ^ab^	44.18 ^a^	0.40	<0.001	0.002	0.008	0.149	Q	<0.001	0.864
C18:2n-6	18.27 ^cd^	20.27 ^a^	18.73 ^b^	18.70 ^bc^	18.12 ^d^	18.37 ^bcd^	0.18	<0.001	0.001	0.001	0.060	Q	<0.001	0.872
C18:3n-3	2.75 ^a^	2.76 ^a^	2.43 ^b^	2.47 ^b^	2.33 ^b^	2.32 ^b^	0.04	<0.001	0.001	0.005	0.046	Q	<0.001	0.761
C20:4n-6	1.45 ^a^	1.41 ^abc^	1.32 ^c^	1.44 ^ab^	1.34 ^c^	1.35 ^bc^	0.01	<0.002	0.134	0.737	0.214	NR	-	-
C20:5n-3	4.00 ^cd^	3.77 ^e^	3.89 ^de^	4.04 ^bc^	4.16 ^ab^	4.25 ^a^	0.04	<0.001	0.001	0.475	0.506	L	<0.001	0.916
C22:2n-6	0.40	0.38	0.37	0.35	0.37	0.36	0.01	>0.2	0.409	0.484	0.564	NR	-	-
C22:6n-3	5.01 ^a^	4.71 ^b^	4.62 ^bc^	4.49 ^cd^	4.42 ^de^	4.35 ^e^	0.05	<0.001	0.001	0.361	0.607	L	<0.001	0.886
∑n-3 HUFA ^3^	9.01 ^a^	8.49 ^b^	8.51 ^b^	8.53 ^b^	8.58 ^b^	8.60 ^b^	0.05	<0.001	0.033	0.929	0.870	L	<0.03	0.294
Unknown	5.57	3.25	3.18	2.97	3.33	3.00	0.23	-	-	-	-	-	-	-

Values (means of triplicate) in the same row sharing the same superscript letter are not significantly different (*p >* 0.05). Abbreviations: SEM, pooled standard error of treatment means; Adj. R^2^, adjusted R square; L, linear; Q, quadratic; C, cubic; NR, no relationship. ^1^ ∑SFA: total content of saturated fatty acids. ^2^ ∑MUFA: total content of monounsaturated fatty acids. ^3^ ∑n-3 HUFA: total content of n-3 highly unsaturated fatty acids.

**Table 8:** Plasma and serum chemistry of rockfish fed experimental diets for 8 weeks.

	Experimental Diets								Orthogonal Contrast			Regression		
	Con	C20J0	C20J20	C20J40	C20J60	C20J80	SEM	*p*-Value	Linear	Quadratic	Cubic	Model	*p-*Value	Adj. R^2^
Plasma parameters														
AST (U/L)	80.1	79.8	84.6	83.0	81.4	81.6	2.15	*>*0.9	0.985	0.718	0.713	NR	-	-
ALT (U/L)	26.1	29.8	30.4	28.2	29.8	27.4	0.69	*>*0.4	0.365	0.740	0.862	NR	-	-
ALP (U/L)	139.1	179.8	179.7	173.2	191.6	157.2	8.09	*>*0.5	0.645	0.609	0.522	NR	-	-
T-BIL (mg/dL)	0.4	0.6	0.4	0.5	0.5	0.5	0.02	>0.2	0.863	0.131	0.188	NR	-	-
T-CHO (mg/dL)	246.7	234.7	244.0	222.9	232.0	238.1	3.53	*>*0.4	0.870	0.516	0.377	NR	-	-
TG (mg/dL)	442.4	446.9	453.3	447.3	451.1	446.2	3.54	*>*0.9	0.914	0.734	0.911	NR	-	-
TP (g/dL)	3.9	4.2	4.2	3.9	4.1	4.2	0.06	*>*0.6	0.793	0.329	0.601	NR	-	-
ALB (g/dL)	0.9	0.9	1.0	0.8	0.9	0.9	0.02	*>*0.4	0.999	0.506	0.331	NR	-	-
Serum parameters														
SOD (ng/mL)	4.0	4.1	4.4	4.0	4.3	3.8	0.24	>0.9	0.748	0.740	0.944	NR	-	-
Lysozyme (U/mL)	301.1	217.3	238.9	251.2	189.7	264.0	18.02	>0.6	0.725	0.833	0.263	NR	-	-

Abbreviations: AST, aspartate aminotransferase; ALT, alanine aminotransferase; ALP, alkaline phosphatase; T-BIL, total bilirubin; T-CHO, total cholesterol; TG, triglyceride; TP, total protein; ALB, albumin; and SOD, superoxide dismutase. SEM, pooled standard error of treatment means; Adj. R^2^, adjusted R square; NR, no relationship.

**Table 9:** Effect of dietary treatments on economic parameters of this study.

	Experimental Diets								Orthogonal Contrast			Regression		
	Con	C20J0	C20J20	C20J40	C20J60	C20J80	SEM	*p*-Value	Linear	Quadratic	Cubic	Model	*p-*Value	Adj. R^2^
Diet price (USD/kg)	1.54	1.43	1.51	1.60	1.69	1.78	-	-	-	-	-	-	-	-
ECR (USD/kg fish) ^1^	1.72 ^b^	1.70 ^b^	1.74 ^b^	1.81 ^ab^	1.89 ^ab^	2.03 ^a^	0.03	<0.01	0.001	0.172	0.886	L	<0.001	0.796
EPI (USD/fish) ^2^	0.256 ^ab^	0.246 ^b^	0.264 ^ab^	0.273 ^ab^	0.291 ^a^	0.285 ^a^	0.005	<0.02	0.002	0.234	0.601	L	<0.001	0.551

Values (means of triplicate) in the same row sharing the same superscript letter are not significantly different (*p >* 0.05). Abbreviations: SEM, pooled standard error of treatment means; Adj. R^2^, adjusted R square; L, linear. ^1^ Economic conversion ratio (ECR, USD/kg) = Feed consumption (kg/fish) × diet price (USD/kg)/weight gain of fish (kg/fish). ^2^ Economic profit index (EPI, USD/fish) = Final weight (kg/fish) × fish sale price (USD/kg) − feed consumption (kg/fish) × diet price (USD/kg).

## Data Availability

Data available on request from the authors.
